# Conservative Quantization of Covariance Matrices with Applications to Decentralized Information Fusion †

**DOI:** 10.3390/s21093059

**Published:** 2021-04-28

**Authors:** Christopher Funk, Benjamin Noack, Uwe D. Hanebeck

**Affiliations:** 1 Intelligent Sensor-Actuator-Systems Laboratory (ISAS), Institute of Anthropomatics and Robotics (IAR), Karlsruhe Institute of Technology (KIT), 76131 Karlsruhe, Germany; uwe.hanebeck@ieee.org; 2 Autonomous Multisensor Systems Group (AMS), Institute for Intelligent Cooperating Systems (ICS), Otto von Guericke University Magdeburg (OVGU), 39106 Magdeburg, Germany; benjamin.noack@ieee.org

**Keywords:** covariance quantization, decentralized estimation, conservative fusion, covariance intersection, optimal fusion

## Abstract

Information fusion in networked systems poses challenges with respect to both theory and implementation. Limited available bandwidth can become a bottleneck when high-dimensional estimates and associated error covariance matrices need to be transmitted. Compression of estimates and covariance matrices can endanger desirable properties like unbiasedness and may lead to unreliable fusion results. In this work, quantization methods for estimates and covariance matrices are presented and their usage with the optimal fusion formulas and covariance intersection is demonstrated. The proposed quantization methods significantly reduce the bandwidth required for data transmission while retaining unbiasedness and conservativeness of the considered fusion methods. Their performance is evaluated using simulations, showing their effectiveness even in the case of substantial data reduction.

## 1. Introduction

Interconnected sensor systems can gather more data, are more robust to faults and outliers, and can cover larger regions than a single sensor system. Such networked systems can also benefit from heterogeneous sensing modalities and parameterizations. Typical examples are wireless sensor networks which are used, for instance, in environmental monitoring [1,2,3], building automation [4,5], or moving object tracking [6,7]. A single node in a wireless sensor network often has limited energy, processing, and storage resources, and the wireless transmission of data is the most energy-intensive operation performed by the node while processing sensor data exhibits relatively low energy demands [8]. Even for networked systems that do not use wireless data transmission or that have sufficient energy resources, communication can be a limiting factor when nodes need to transmit large-scale estimates, which may occur in cooperative map building [9], cooperative localization [10,11], or multi-object tracking [12].

From the accruing sensor data, the interconnected devices can compute state estimates locally, e.g., by employing Kalman filter methods. Such estimates are typically supplied with error covariance matrices, which need to be transmitted and stored alongside the estimates, to be able to assess their uncertainty and combine them reliably. Therefore, reducing the amount of transmitted data through prior compression is key to meeting bandwidth limitations when high-dimensional state estimates are exchanged and to ensure long operating times of battery-driven sensor nodes when wireless data transmission is used. A comprehensive survey of lossless and lossy compression methods that are suitable for wireless sensor networks is given in [13]. The surveyed methods include multiple probabilistic quantization-based approaches [14,15,16,17] tailored to estimation problems. However, only scalar estimates are considered and their respective variances are assumed to be known to the receiver. Quantization as a means of data reduction has also been applied to Kalman filtering, a prominent example being the sign of innovations Kalman filter [18,19]. Again, the required covariance matrices are assumed to be known to the receiver. In contrast to the previous works, it is assumed in [20] that the receiver has no prior knowledge of the covariance matrices, which therefore need to be transmitted via the network. The authors develop data reduction methods for covariance matrices based on conservative diagonal approximations and, in [21], they investigate techniques to select subsets of the information to be transmitted. These methods assess how the selected information contributes to the receiver’s estimation quality.

In this paper, similarly to [20], we assume that the receiver has no knowledge about the covariance matrix at the transmitter. Hence, the sender has to prepare both its estimate and covariance matrix for transmission. In general, the covariance matrix will not be diagonal and dominates the amount of data that needs to be transmitted as its number of elements grows quadratically with the dimension of the estimate. The individual quantization of each coefficient constitutes a promising approach to reduce the data. However, such a quantized covariance matrix, in general, does not reliably account for the uncertainty of the estimate and can even violate the positive definiteness of the covariance matrix. For this reason, we study and compare two different approaches to compute a quantized covariance matrix that conservatively bounds the actual error covariance matrix. The first scheme employs a quantization based on diagonal dominance while the second scheme relies on a modified Cholesky decomposition. To further compress the data to be transmitted, we also investigate a quantization of the estimates. As typical fusion algorithms rely on unbiasedness, we employ a quantizer that preserves this property. The proposed quantization schemes yield conservative estimates that reliably assess the estimation error and can be further processed at the receiver.

At the receiver, the estimates are typically fed to a fusion algorithm to combine them with other estimates and to improve the estimation accuracy. Fusion algorithms that strive to minimize the error of the fusion result need access to the covariance matrices of the input estimates. Optimal fusion algorithms [22,23] can be designed if cross-correlations between the estimates are also known. They typically require the transmission of additional information [24] or specific communication strategies [25,26]. In the case where correlations are unknown, conservative fusion algorithms compute a bound on the actual but unknown error covariance matrix of the fusion results. Examples of such algorithms are covariance intersection (CI) [27], fast covariance intersection (FCI) [28,29], and inverse covariance intersection (ICI) [30,31], which are guaranteed to produce results with a conservative uncertainty quantification in the form of a covariance matrix. Other algorithms such as ellipsoidal intersection (EI) [32,33] provide no such guarantee but are typically less conservative. In this paper, we study how the proposed quantization schemes integrate with fusion algorithms and consider both optimal fusion and covariance intersection.

This paper is an extended version of [34], which proposed a quantization technique for covariance intersection. Here, we study the use of quantization in a broader sense to cover different fusion algorithms, and we propose an additional quantization scheme for covariance matrices that provides tighter bounds at the expense of higher computational demand. In total, this paper’s contributions address three different aspects:

**Estimate Quantization.** We extend the probabilistic quantization method from [15,35] to vector-valued correlated random variables in order to generate unbiased quantized estimates [34] and conservative bounds on their error covariance matrices.
**Covariance Quantization.** We propose two approaches to the conservative quantization of covariance matrices. The first scheme uses diagonal dominance [34]. As an alternative, we study a modified Cholesky decomposition and compare it to the first approach.
**Fusion of Estimates.** We apply the quantization schemes to both an optimal fusion algorithm and covariance intersection in order to demonstrate that reliable estimates are attained.

Implementations of the proposed quantization schemes, written in Python, are provided as supplementary material, the link to which can be found at the end of the paper.

## 2. Notation

Lower case letters x∈R denote scalar quantities and additional underlining $\underline{x}\in R^{n}$ indicates *n*-dimensional vector-valued quantities. The standard basis vectors of $R^{n}$ are ${\underline{e}}_{1},\ldots ,{\underline{e}}_{n}$ and ${\underline{0}}_{n}$ denotes the *n*-dimensional zero vector. Bold uppercase letters indicate n×n-matrices such as $X\in R^{n\times n}$. The *i*th coefficient of a vector and the i,jth coefficient of a matrix are ${\underline{x}}^{i}$ and $X^{i,j}$, respectively. In addition, index ranges such as i:j, i:, and :i are used to extract subvectors and submatrices. As an example, ${\underline{x}}^{i:j}$ is the subvector containing the *i*th to the *j*th coefficient of $\underline{x}$ and $X^{:i,j}$ are the first *i* coefficients of the *j*th column of X. The use of boldface as in x∈R and $\underline{x}\in R^{n}$ indicates random scalars and random vectors, respectively. Uppercase calligraphic letters A indicate sets. In particular, $S^{n}$ is used to denote the set of symmetric matrices in $R^{n\times n}$ and $S_{+}^{n}$ to denote the set of symmetric positive semi-definite (PSD) matrices in $R^{n\times n}$. For $X\in S_{+}^{n}$ and $Y\in S_{+}^{n}$, the notation X⪯Y signifies that $Y-X\in S_{+}^{n}$. If X⪯Y then $Y\in S_{+}^{n}$ is called an upper bound for $X\in S_{+}^{n}$. For (conditional) expectations the symbols E(·) and E(·|·) are used. The unconditional covariance between two random quantities is designated by C(·,·) or by C(·) if the arguments are identical. Similarly, the conditional covariance is denoted by C(·,·|·) or C(·|·).

## 3. Considered Problem

The process of quantization approximates a continuous quantity using a discrete one. In this work, we consider the quantization of covariance matrices and estimates with the goal of reducing the bandwidth and storage requirements on an interconnected sensor system. We demonstrate how optimal fusion and covariance intersection can be applied to quantized data while retaining some of their desirable properties.

**Figure 1:**
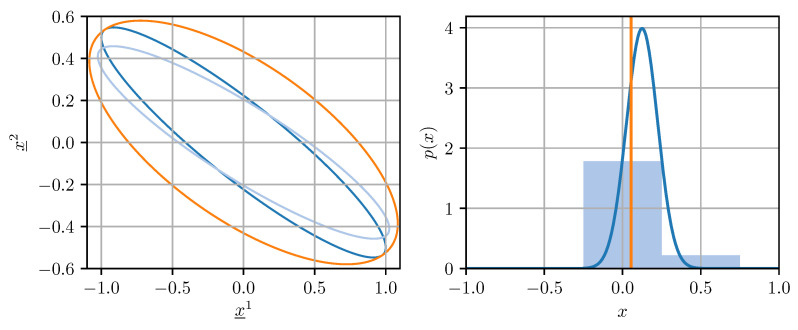
(**Left**): Confidence ellipsoids of a covariance matrix (dark blue), its naively quantized version (light blue), and its conservatively quantized version (orange). (**Right**): Density of a Gaussian random variable (dark blue), histogram (light blue), and mean (orange) of its quantized version.

For our purposes, a quantizer is a map q:D→C, where the domain D is a closed, coefficient-wise bounded subset of either $R^{n}$ or $R^{n\times n}$, and the codomain C, the so-called codebook, is a finite set. Quantizing covariance matrices for use in fusion methods is not straightforward. Naive coefficient-wise quantization of a covariance matrix can lead to a result that underestimates the uncertainty encoded in the original covariance matrix, or even worse, is not a valid covariance matrix anymore. This can cause divergence in certain estimation algorithms [27]. Ideally, the quantized covariance matrix q(X) should be an upper bound on a conservative estimate of the original matrix X in the sense that q(X)⪰X holds. This averts divergence and guarantees that the confidence ellipsoid induced by q(X) contains the one induced by X [27]. The described situation is illustrated on the left side of Figure 1. Conservative quantization of covariance matrices can be achieved by enforcing certain conditions on the quantization error, as will be discussed in Section 4.

Similarly to quantizing covariance matrices, quantizing estimates for use in fusion methods creates certain challenges. Deterministic quantization of an estimate can introduce bias and additional noise, which (1) biases the results obtained from fusion methods and (2) invalidates the covariance matrix associated with the estimate. This is visualized on the right side of Figure 1. Both of the aforementioned issues can be addressed by using randomized quantizers, which will be discussed in Section 5 in the context of applying fusion methods to quantized data.

## 4. Conservative Quantization of Covariance Matrices

In the following, conservative quantizers for covariance matrices, i.e., symmetric positive semi-definite (PSD) matrices, are derived. To that end, let $q_{c}:D_{c}\to C_{c}$ be a quantizer that maps PSD matrices from an coefficient-wise bounded and closed set $D_{c}\subset S_{+}^{n}$ to a finite codebook $C_{c}\subset R^{n\times n}$. The quantizer $q_{c}$ should satisfy the condition

(1) $$\begin{array}{cc} \forall X\in D_{c}:q_{c}\left(X\right) & ⪰X\;, \end{array}$$

to ensure that the quantized matrix $q_{c}\left(X\right)$ is an upper bound on the original matrix X. With the quantization error defined as $\Delta \left(X\right)=q_{c}\left(X\right)-X$, this can also be expressed as

(2) $$\begin{array}{cc} \forall X\in D_{c}:\Delta \left(X\right) & ⪰0\;. \end{array}$$

In other words, the quantization error must always be PSD for the quantized matrix to be an upper bound on the original matrix. Ideally, the quantizer should not only produce conservative results but should also minimize the total quantization error ${∥\Delta \left(X\right)∥}_{F}$. This requires enumerating all elements in $C_{c}$ in the worst case and is thus not computationally feasible, even for relatively small matrices. Practical quantizers will therefore not be able to minimize the total quantization error exactly. To remain computationally tractable, the quantizers considered in this work operate in two steps: First, the off-diagonal coefficients are individually rounded to the nearest codeword in some off-diagonal codebook $C_{o}\subset R$. Then, the diagonal coefficients are individually rounded up to a codeword in some diagonal codebook $C_{d}\subset R$ so as to make the quantization error PSD.

### 4.1. Covariance Quantization Based on Diagonal Dominance

The rounding method for diagonal coefficients considered in this section is based on the notion of diagonal dominance. Diagonal dominance is a simple sufficient condition for a symmetric matrix, such as the quantization error matrix Δ(X), to be PSD. A symmetric matrix $X\in S^{n}$ is said to be diagonally dominant if

(3) $$X^{i,i}\geq \sum_{j=1\neq i}^{n}\left|X^{i,j}\right|$$

holds for each row i=1,…,n. The connection between diagonal dominance and positive semi-definiteness is obtained immediately by applying the Gershgorin circle theorem [36] to a diagonally dominant matrix to lower bound its eigenvalues.

**Theorem** **1.** *Let $X\in S^{n}$ be diagonally dominant, then X⪰0 holds.*

The approach to conservative quantization of a PSD matrix $X\in D_{c}$ pursued here is to first quantize the off-diagonal coefficients of X using a codebook $C_{o}\subset R$ and to then quantize the diagonal coefficients using a codebook $C_{d}\subset R$ such that (3) is satisfied for the quantization error Δ(X). This leads to the quantizer

(4) $$\begin{array}{cc} q_{c}{\left(X\right)}^{i,j} & =\left\{\begin{array}{cc} \left\lceil X^{i,j}+\sum_{k=1\neq i}^{n}\left|\mathrm{rd}\left(X^{i,k}\right)-X^{i,k}\right|\right\rceil \;, & i=j\\ \mathrm{rd}(X^{i,j})\;, & i\neq j \end{array}\right.\;, \end{array}$$

where rd(·) rounds to the nearest codeword in the off-diagonal codebook $C_{o}$ and ⌈·⌉ rounds up to the nearest codeword in the diagonal codebook $C_{d}$. The codebooks are

(5) $$\begin{array}{cc} C_{o} & =\{x_{max}-k\delta_{o}\;|\;0\leq k<2^{b}\}\;, \end{array}$$

(6) $$\begin{array}{cc} C_{d} & =\{x_{max}+\left(n-1\right)\delta_{o}/2-k\delta_{d}\;|\;0\leq k<2^{b}\}\;, \end{array}$$

where $x_{max}$ is the maximum off-diagonal codeword, and $\delta_{o}=x_{max}/2^{b-1}$, $\delta_{d}=\left(x_{max}+\left(n-1\right)\delta_{o}/2\right)/\left(2^{b}-1\right)$ are quantization resolutions, with *b* the number of bits per codeword. This choice enables the following theorem regarding well definedness.

**Theorem** **2.** *The quantizer $q_{c}:D_{c}\to C_{c}$ proposed above is well defined if the coefficients of all matrices $X\in D_{c}$ are contained in the interval $\left[\min\left(C_{o}\right),\max\left(C_{o}\right)\right]$.*

**Proof.**  The quantization error of an off-diagonal coefficient is $\delta_{o}/2$ at most. Therefore

$$\begin{array}{c} X^{i,i}+\sum_{k=1\neq i}^{n}\left|\mathrm{rd}\left(X^{i,k}\right)-X^{i,k}\right|\leq \max\left(C_{o}\right)+\left(n-1\right)\frac{\delta_{o}}{2} \end{array}$$

holds for all diagonal coefficients. Since the right hand side equals $\max(C_{d})$, rounding up the perturbed diagonal coefficients is always possible, and the claim holds. □

When not stated otherwise, the above conditions for well defined $q_{c}$ are implicitly assumed to hold. The next theorem confirms that the output of $q_{c}$ is indeed an upper bound for its input.

**Theorem** **3.** *The quantizer $q_{c}:D_{c}\to C_{c}$ proposed above has PSD quantization error $\Delta \left(X\right)=q_{c}\left(X\right)-X$ for all $X\in D_{c}$ and is thus conservative, that is, $q_{c}\left(X\right)⪰X$ holds for all $X\in D_{c}$.*

**Proof.**  In the following, we omit the dependence of Δ(X) on X for brevity. The off-diagonal quantization errors are $\Delta^{i,j}=\mathrm{rd}\left(X^{i,j}\right)-X^{i,j}$ and the diagonal ones are

$$\begin{array}{cc} \Delta^{i,i} & =\left\lceil X^{i,i}+\sum_{j=1\neq i}^{n}\left|\Delta^{i,j}\right|\right\rceil -X^{i,i}\;. \end{array}$$

By the definition of ⌈·⌉ we have

$$\begin{array}{cc} \Delta^{i,i} & \geq \sum_{j=1\neq i}^{n}\left|\Delta^{i,j}\right| \end{array}$$

and the claim follows from Theorem 1. □

Furthermore, the quantizer $q_{c}$ introduced above is optimal in the sense that, given codebooks $C_{d/o}$, there is no quantizer with symmetric diagonally dominant quantization error Δ(X) that has a smaller total quantization error ${∥\Delta \left(X\right)∥}_{F}$.

**Theorem** **4.** *Let $q_{c}:D_{c}\to C_{c}$ be defined by (4) with coefficient-wise codebooks $C_{d}$ and $C_{o}$ defined by (5) and (6). Given $X\in D_{c}$, the quantization error $\Delta \left(X\right)=q_{c}\left(X\right)-X$ is the minimizer of*

(7) $$\begin{array}{cccccc} \underset{\Delta \in S^{n}}{\mathrm{minimize}} & \; & & {∥\Delta ∥}_{F}^{2} & \; & \end{array}$$

(8) $$\begin{array}{cccccc} \mathrm{subject}\;\mathrm{to} & & & \sum_{j=1\neq i}^{n}\left|\Delta^{i,j}\right|\leq \Delta^{i,i} & & \forall i=1\ldots n \end{array}$$

(9) $$\begin{array}{cccccc} & & & X^{i,i}+\Delta^{i,i}\in C_{d} & & \forall i=1\ldots n \end{array}$$

(10) $$\begin{array}{cccccc} & & & X^{i,j}+\Delta^{i,j}\in C_{o} & & \forall i\neq j \end{array}$$

*where the dependency of Δ(X) on X has been omitted for brevity.*

**Proof.**  The problem can be reformulated as a nested minimization, the inner one

$$\begin{array}{cccccc} \underset{\Delta^{i,i}\in R,\;i=1,\ldots ,n}{\mathrm{minimize}} & \; & & \sum_{i=1}^{n}{\left|\Delta^{i,i}\right|}^{2} & \; & \\ \mathrm{subject}\;\mathrm{to} & & & \sum_{j=1\neq i}^{n}\left|\Delta^{i,j}\right|\leq \Delta^{i,i} & & \forall i=1\ldots n\\ & & & X^{i,i}+\Delta^{i,i}\in C_{d} & & \forall i=1\ldots n \end{array}$$

being over the diagonal coefficients given the off-diagonal coefficients and the outer one

$$\begin{array}{cccccc} \underset{\Delta^{i,j}\in R,i\neq j}{\mathrm{minimize}} & \; & & \sum_{i=1}^{n}|\Delta^{i,i*}{|}^{2}+\sum_{i=1}^{n}\sum_{j=1\neq i}^{n}{\left|\Delta^{i,j}\right|}^{2} & \; & \\ \mathrm{subject}\;\mathrm{to} & & & X^{i,j}+\Delta^{i,j}\in C_{o} & & \forall i\neq j \end{array}$$

being over the off-diagonal coefficients given the solutions $\Delta^{i,i*}$ of the inner optimization. Furthermore, the inner minimization can be split into decoupled minimizations

$$\begin{array}{cccccc} \underset{\Delta^{i,i}\in R}{\mathrm{minimize}} & \; & & |\Delta^{i,i}{|}^{2} & \; & \\ \mathrm{subject}\;\mathrm{to} & & & \sum_{j=1\neq i}^{n}\left|\Delta^{i,j}\right|\leq \Delta^{i,i} & & \\ & & & X^{i,i}+\Delta^{i,i}\in C_{d} & \end{array}$$

for i=1,…,n. By definition of the ⌈·⌉ operation

$$\begin{array}{cc} \Delta^{i,i*} & =\left\lceil X^{i,i}+\sum_{j=1\neq i}^{n}\left|\Delta^{i,j}\right|\right\rceil -X^{i,i} \end{array}$$

are the optimal solutions to these subproblems. They exist because $q_{c}$ is well defined. The minimum cost of each decoupled problem is thus

$$\begin{array}{cc} |\Delta^{i,i*}{|}^{2} & ={\left|\left\lceil X^{i,i}+\sum_{j=1\neq i}^{n}\left|\Delta^{i,j}\right|\right\rceil -X^{i,i}\right|}^{2}\;, \end{array}$$

which is non-decreasing in each $\left|\Delta^{i,j}\right|$. Using this intermediate result, the outer minimization problem can be seen to attain its minimum by separately minimizing the $|\Delta^{i,j}{|}^{2}$, as due to the non-decreasing property, $|\Delta^{i,i*}{|}^{2}$ is minimal if each $|\Delta^{i,j}{|}^{2}$ is minimal. Thus, the minimum is, by definition of rd(·), attained by setting $\Delta^{i,j*}=\mathrm{rd}\left(X^{i,j}\right)$. □

Although the above quantizer minimizes the conservativeness of the quantized matrix in the sense of Theorem 4, the inequalities (3) are only sufficient and not necessary for the quantization error to be PSD. Hence, the results of this method are usually more conservative than necessary. The proposed quantizer has a low computational complexity of $O(n^{2})$ because the matrix coefficients are quantized individually.

### 4.2. Covariance Quantization Based on Modified Cholesky Decomposition

The covariance matrix quantization approach presented in the previous section is computationally efficient but can be overly conservative. An alternative quantizer that is guaranteed to be less conservative at the cost of increased computational expense is presented in this section. The basic approach of first quantizing the off-diagonal coefficients and then finding quantized diagonal coefficients that make the overall quantization result conservative is retained. However, instead of employing diagonal dominance to find the quantized diagonal coefficients, a modified Cholesky factorization adopted from [37] is leveraged. We motivate the proposed method by first introducing the Cholesky decomposition in conjunction with a result relating its existence to positive semi-definiteness [36] (Corollary 7.2.9).

**Theorem** **5.** *Let $X\in S^{n}$ be a symmetric matrix and $P\in R^{n\times n}$ a permutation matrix (Permutation matrices are orthogonal matrices that arise by permuting the rows and columns of an identity matrix. Matrix multiplication with a permutation matrix permutes either the rows or the columns of the other matrix, depending on the order of multiplication). Then there is a lower triangular matrix $L\in R^{n\times n}$ with nonnegative diagonal coefficients such that*

(11) $$\begin{array}{cc} PXP^{⊤} & =LL^{⊤} \end{array}$$

*holds if and only if X is positive semi-definite. The above factorization is called a (pivoted) Cholesky decomposition of X with Cholesky factor L.*

Should X not be PSD, a so-called modified Cholesky decomposition can be performed to find a diagonal nonnegative matrix D such that a Cholesky decomposition $P\left(X+D\right)P^{⊤}=LL^{⊤}$ exists [37,38,39]. In the following, the basic recursive approach to simultaneously compute the matrices D, P, and L is introduced, based on the exposition in [37]. The recursion begins by setting $X_{1}=X$. The computations

(12) $$\begin{array}{cc} {\bar{X}}_{k} & =P_{k}X_{k}P_{k}^{⊤}+s_{k}{\underline{e}}_{k}{\underline{e}}_{k}^{⊤} \end{array}$$

(13) $$\begin{array}{cc} X_{k+1} & ={\bar{X}}_{k}-{\underline{x}}_{k}{\underline{x}}_{k}^{⊤} \end{array}$$

are then performed for k=1,…,n. Each $P_{k}$ is a permutation matrix swapping two rows and columns such that ${\left(P_{k}X_{k}P_{k}^{⊤}\right)}^{k,k}=X_{k}^{m,m}$ with m≥k determined according to some criterion. For now we will assume m=k so that $P_{k}=I$. The $s_{k}$ are nonnegative perturbations applied to the *k*th diagonal coefficient of $P_{k}X_{k}P_{k}^{⊤}$. The vector ${\underline{x}}_{k}\in R^{n}$ is selected to cancel the *k*th row and column of ${\bar{X}}_{k}$. This is achieved by letting

(14) $${\underline{x}}_{k}^{⊤}=\left\{\begin{array}{cc} {\underline{0}}_{n}^{⊤} & ,\;{\bar{X}}_{k}^{k,k:}={\underline{0}}_{n-k+1}^{⊤}\\ \left[\begin{array}{ccc} {\underline{0}}_{k-1}^{⊤} & \sqrt{{\bar{X}}_{k}^{k,k}} & \frac{{\bar{X}}_{k}^{k,k+1:}}{\sqrt{{\bar{X}}_{k}^{k,k}}} \end{array}\right] & ,\;\mathrm{else} \end{array}\right.$$

and results in the k−1 upper-most/left-most rows and columns of $X_{k}$ being zero. For the recursion to terminate successfully, $s_{k}$ must either be such that ${\bar{X}}_{k}^{k,k}$ is positive or such that ${\bar{X}}_{k}^{k,k:}$ is zero. This is always possible, as $s_{k}$ can be arbitrarily large. Unraveling the recursion up to $X_{n+1}$ and using the fact that $X_{n+1}=0$ gives

(15) 0=∏k=1nPn−k+1X+∑k=1nsk∏j=1kPj⊤e_k∏j=1kPj⊤e_k⊤∏k=1nPn−k+1⊤=−∑k=1n∏j=1n−kPn−j+1x_k∏j=1n−kPn−j+1x_k⊤.

This can be written in the more condensed form

(16) $$\begin{array}{cc} 0 & =P\left(X+D\right)P^{⊤}-LL^{⊤} \end{array}$$

by introducing $P=\prod_{k=1}^{n}P_{n-k+1}$, $L=\left[\begin{array}{ccc} {\underline{l}}_{1} & \cdots & {\underline{l}}_{n} \end{array}\right]$ with ${\underline{l}}_{k}=\prod_{j=1}^{n-k}P_{n-j+1}\;{\underline{x}}_{k}$, and

(17) $$D=\sum_{k=1}^{n}s_{k}\left(\prod_{j=1}^{k}P_{j}^{⊤}{\underline{e}}_{k}\right){\left(\prod_{j=1}^{k}P_{j}^{⊤}{\underline{e}}_{k}\right)}^{⊤}.$$

It can be shown that P is a permutation matrix, L is lower triangular with nonnegative diagonal coefficients, and D has its diagonal populated with the $s_{k}$ and is otherwise zero. Hence, (16) is a Cholesky decomposition of X+D and according to Theorem 5, X+D must be positive semi-definite. Note that the above recursion can be computed in-place essentially like an ordinary Cholesky decomposition (see for instance [40] (Algorithm 4.2.2)), the main differences being the diagonal shifts $s_{k}$ and allowing ${\underline{x}}_{k}={\underline{0}}_{n}$.

We will now describe how the above approach can be applied to finding quantized diagonal coefficients that make the overall quantization result conservative. For that, first quantize each off-diagonal coefficient of the given PSD matrix $X\in D_{c}$ by rounding it to the nearest codeword in $C_{o}=\left\{x_{max}-k\delta_{o}\;|\;0\leq k<2^{b}\right\}$ (see Section 4.1) resulting in the preliminary quantized matrix and preliminary quantization error

(18) $$\begin{array}{cccc} X_{o}\left(X\right) & =\left\{\begin{array}{cc} X^{i,j}\;, & i=j\\ \mathrm{rd}(X^{i,j})\;, & i\neq j \end{array}\right.\;, & \Delta_{o}\left(X\right) & =X_{o}-X\;. \end{array}$$

Note that in the remainder of this section we will omit the dependence of $X_{o}\left(X\right)$ and $\Delta_{o}\left(X\right)$ on X for brevity. Since the diagonal elements of $\Delta_{o}$ are zero, $\Delta_{o}$ cannot be PSD unless it is zero, as can be easily verified. Then the modified Cholesky decomposition of $\Delta_{o}$ is computed, giving a diagonal matrix D such that $\Delta_{o}+D⪰0$ holds. Adding D to $X_{o}$ and rounding the diagonal coefficients up to the nearest codeword in $C_{d}=\left\{x_{max}+\left(n-1\right)\delta_{o}/2-k\delta_{d}\;|\;0\leq k<2^{b}\right\}$ (see Section 4.1) then gives the final quantization result

(19) $$\begin{array}{cc} q_{c}\left(X\right) & =\left\{\begin{array}{cc} \left\lceil X_{o}^{i,j}+D^{i,j}\right\rceil \;, & i=j\\ X_{o}^{i,j}\;, & i\neq j \end{array}\right.\;. \end{array}$$

Assume for the moment that the quantizer defined above is well-defined, i.e., that there always are codewords in $C_{d}$ that the perturbed diagonal elements can be rounded up to. In that case, the following theorem applies.

**Theorem** **6.** *The quantizer $q_{c}:D_{c}\to C_{c}$ as defined above is conservative.*

**Proof.**  Due to the modified Cholesky decomposition, it holds that $X_{o}-X+D=\Delta_{o}+D⪰0$. By rounding the diagonal of $X_{o}+D$ up, we get $X_{o}+D+\Delta_{d}$ where $\Delta_{d}$ is diagonal and nonnegative and thus $\Delta_{d}⪰0$. Therefore, $X_{o}+D+\Delta_{d}-X⪰X_{o}+D-X⪰0$ or equivalently $q_{c}\left(X\right)⪰X$ holds. □

So far, the diagonal perturbations $s_{k}$ have been assumed to be almost arbitrary. In order to guarantee that the quantizer is well-defined, we adopt the specific choice

(20) $$\begin{array}{c} s_{k}=\max\left\{0,{∥{\left(P_{k}X_{k}P_{k}^{⊤}\right)}^{k+1:,k}∥}_{1}-{\left(P_{k}X_{k}P_{k}^{⊤}\right)}^{k,k}\right\}, \end{array}$$

described in [37]. This has several advantageous implications as can be seen from Theorem 7 [37] (Theorem 5.1.2) and the subsequent corollaries.

**Theorem** **7.** *If $s_{k}$ is chosen as in (20) to compute the modified Cholesky decomposition of some $X\in S^{n}$, then X+D is positive semi-definite (it can be rank-deficient) and the upper bound*

(21) $$s_{k}\leq \max\left\{0,\max_{j}\left(-X^{j,j}+\sum_{i=1\neq j}^{n}\left|X^{i,j}\right|\right)\right\}$$

*holds for k=1,…,n. The result is valid, provided each $P_{k}$ swaps the kth row and column only with some subsequent row and column.*

Applying the above theorem to the quantizer proposed in this section, it is evident that with the given choice of $s_{k}$, the diagonal perturbations are always smaller than or equal to the maximum perturbation required by the quantization approach from Section 4.1. Inspecting the proof in [37], it can indeed be deduced that every $s_{k}$ is smaller than or equal to its corresponding perturbation (after permutation using the $P_{k}$) in the diagonal dominance based approach.

**Corollary** **1.** *The quantizer $q_{c}:D_{c}\to C_{c}$ proposed in this section is well defined if the coefficients of all matrices in $D_{c}$ are in the interval $\left[\min\left(C_{o}\right),\max\left(C_{o}\right)\right]$.*

**Proof.**  By applying Theorem 7 to $\Delta_{o}$, it follows that $s_{k}\leq \max_{j}\sum_{i=1\neq j}^{n}\left|\Delta_{o}^{i,j}\right|$ holds for k=1,…,n. Comparing to (4), $s_{k}$ can be seen to be smaller than the maximum amount added to diagonal coefficients by the quantizer from Section 4.1. Hence, following the same argument as for Theorem 2 and using the bound on $s_{k}$, the claim follows. □

**Corollary** **2.** *The quantizer $q_{c}:D_{c}\to C_{c}$ proposed in this section has lower or identical total quantization error ${∥\Delta ∥}_{F}$ compared to the quantizer from Section 4.1.*

**Proof.**  By the discussion above, the diagonal perturbations $s_{k}$ are smaller than or equal to those used by the quantizer based on diagonal dominance. This translates into a reduced absolute deviation from the original diagonal elements, also after rounding up. The off-diagonal quantization errors are identical. Hence, the total quantization error is less than or equal to that of the quantizer based on diagonal dominance. □

The final ingredient in the above quantization approach is the choice of the permutation matrices $P_{k}$ which, up to now, have been assumed to be identity matrices. We adopt the choice of $P_{k}$ proposed in [37], which greatly improved performance in our experiments. Each matrix $P_{k}$ is chosen in order to swap two rows and columns such that ${\left(P_{k}X_{k}P_{k}^{⊤}\right)}^{k,k}=X_{k}^{m,m}$ with $m=\arg\max_{k\leq i\leq n}{\underline{g}}_{k}^{i}$ where ${\underline{g}}_{k}$ is a recursively computed vector initialized at k=1 using

(22) $$\begin{array}{cc} {\underline{g}}_{1}^{i} & =X^{i,i}-\sum_{j=1\neq i}^{n}\left|X^{i,j}\right|\;,i=1,\ldots ,n \end{array}$$

and recursively updated for each k=1,…,n according to

(23) $$\begin{array}{cc} {\underline{g}}_{k+1}^{i} & ={\left(P_{k}{\underline{g}}_{k}\right)}^{i}+\left|{\left(P_{k}X_{k}P_{k}^{⊤}\right)}^{k,i}\right|\left(1-\frac{∥{\left(P_{k}X_{k}P_{k}^{⊤}\right)}^{k,k+1:}{∥}_{1}}{{\bar{X}}_{k}^{k,k}}\right)\;,i=k+1,\ldots ,n \end{array}$$

after choosing the respective $P_{k}$ and $s_{k}$. These changes do not affect the theoretical results, as they only pertain to the strategy of choosing $P_{k}$. We do not take into account the remaining modifications proposed in [37], as they do not seem to improve performance in our case. Due to the modified Cholesky decomposition, the quantizer introduced in this section has computational complexity of $O(n^{3})$, compared to the $O(n^{2})$ complexity of the quantizer from Section 4.1.

## 5. Applications to Information Fusion

The goal of any fusion algorithm is to combine estimates ${\underline{x}}_{a}\in R^{n}$ and ${\underline{x}}_{b}\in R^{n}$ of the same random quantity $\underline{x}\in R^{n}$ to obtain an, in some sense, improved estimate ${\underline{x}}_{f}\in R^{n}$ of $\underline{x}$. Typically, the estimates ${\underline{x}}_{a}$ and ${\underline{x}}_{b}$ are provided in conjunction with error covariance matrix estimates $C_{aa}\approx C\left({\underline{x}}_{a}-\underline{x}\right)$ and $C_{bb}\approx C\left({\underline{x}}_{b}-\underline{x}\right)$ and the fusion method uses them to compute an error covariance estimate $C_{ff}\approx C\left({\underline{x}}_{f}-\underline{x}\right)$ for the fused estimate ${\underline{x}}_{f}$.

In the following, two fusion methods from the literature that are unbiased ($E\left({\underline{x}}_{f}\right)=E\left(\underline{x}\right)$) and conservative ($C_{ff}⪰C\left({\underline{x}}_{f}-\underline{x}\right)$) under certain conditions, are introduced and their application with quantized error covariance matrices and estimate vectors is considered. In that context, a quantizer for unbiased estimate vectors is derived, that retains their unbiasedness and provides a conservative estimate of the error covariance matrix of the quantization result. Finally, it is demonstrated how the covariance quantization methods from Section 4 can be applied in conjunction with the unbiased estimate quantizer to retain unbiasedness and conservativeness of the fusion methods.

### 5.1. Optimal Fusion and Covariance Intersection

In the following, let ${\underline{x}}_{a}\in R^{n}$ and ${\underline{x}}_{b}\in R^{n}$ be unbiased estimates of some random vector $\underline{x}\in R^{n}$ and let

(24) $$C_{cc}=C\left(\left[\begin{array}{c} {\underline{x}}_{a}-\underline{x}\\ {\underline{x}}_{b}-\underline{x} \end{array}\right]\right)=\left[\begin{array}{cc} C_{aa} & C_{ab}\\ C_{ba} & C_{bb} \end{array}\right]$$

be their joint error covariance matrix. The fused estimate and its associated estimated error covariance matrix will be denoted by ${\underline{x}}_{f}$ and $C_{ff}$, respectively. Note that $C_{ff}$ need not necessarily be identical to the actual error covariance matrix $C({\underline{x}}_{f}-\underline{x})$. In case the cross-covariance $C_{ab}$ is known, the optimal fusion result in the BLUE (best linear unbiased estimator) sense is given by the Bar-Shalom–Campo formulas [22]

(25) $$\begin{array}{cc} {\underline{x}}_{f} & ={\underline{x}}_{a}+\left(C_{aa}-C_{ab}\right){\left(C_{aa}+C_{bb}-C_{ab}-C_{ba}\right)}^{-1}\left({\underline{x}}_{b}-{\underline{x}}_{a}\right)\;, \end{array}$$

(26) $$\begin{array}{cc} C_{ff} & =C_{aa}-\left(C_{aa}-C_{ab}\right){\left(C_{aa}+C_{bb}-C_{ab}-C_{ba}\right)}^{-1}\left(C_{aa}-C_{ba}\right)\;. \end{array}$$

The estimated error covariance matrix computed using the Bar-Shalom–Campo formulas is exact, i.e., $C_{ff}=C\left({\underline{x}}_{f}-\underline{x}\right)$ holds [22]. The cross-covariance $C_{ab}$ required by (25) and (26) can be tracked, e.g., using samples [41] to encode the cross-correlations or by a square-root decomposition [24] of the noise covariance matrices. Both approaches require additional data to be transmitted. If the error covariance matrices $C_{aa}$ and $C_{bb}$ are not known, but upper bounds ${\hat{C}}_{aa}⪰C_{aa}$ and ${\hat{C}}_{bb}⪰C_{bb}$ are available, applying (25) and (26) using the estimates yields a conservative error covariance matrix estimate $C_{ff}⪰C\left({\underline{x}}_{f}-\underline{x}\right)$ [42].

**Figure 2:**
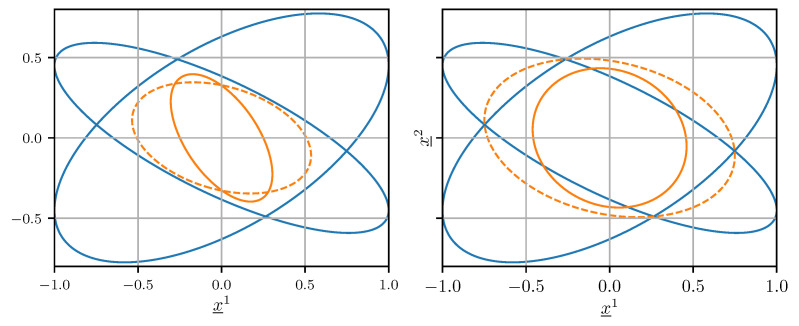
Confidence ellipsoids of $C_{aa}$ and $C_{bb}$ (blue), $C({\underline{x}}_{f}-\underline{x})$ (orange, solid), and $C_{ff}$ (orange, dashed) when $C_{ab}\neq 0$. The result obtained using optimal fusion with the erroneous assumption $C_{ab}=0$ is shown on the left. The result achieved using CI is shown on the right.

In the common case where the cross-covariance is unknown but not negligible, setting $C_{ab}=0$ in (25) and (26) generally does not produce a conservative error covariance matrix estimate, i.e., $C_{ff}C\left({\underline{x}}_{f}-\underline{x}\right)$. This means that the confidence ellipsoid induced by $C_{ff}$ does not contain the confidence ellipsoid induced by $C({\underline{x}}_{f}-\underline{x})$ for any ϵ>0. An example of this behavior is illustrated on the left side of Figure 2.

The covariance intersection (CI) algorithm, originally devised by Julier and Uhlmann [27], enables the conservative and unbiased fusion of the estimates ${\underline{x}}_{a}$ and ${\underline{x}}_{b}$, regardless of their generally unknown cross-covariance, as long as conservative error covariance matrix estimates ${\hat{C}}_{aa}⪰C_{aa}$ and ${\hat{C}}_{bb}⪰C_{bb}$ are available. The CI algorithm itself is defined by

(27) $$\begin{array}{cc} C_{ff}^{-1} & =\omega {\hat{C}}_{aa}^{-1}+\left(1-\omega \right){\hat{C}}_{bb}^{-1}\;, \end{array}$$

(28) $$\begin{array}{cc} C_{ff}^{-1}{\underline{x}}_{f} & =\omega {\hat{C}}_{aa}^{-1}{\underline{x}}_{a}+\left(1-\omega \right){\hat{C}}_{bb}^{-1}{\underline{x}}_{b}\;, \end{array}$$

where any ω∈[0,1] gives an unbiased estimate ${\underline{x}}_{f}$ and an upper bound $C_{ff}$ on its error covariance matrix $C({\underline{x}}_{f}-\underline{x})$. The weight ω is determined numerically by minimizing either the trace or the determinant of $C_{ff}$. The right side of Figure 2 shows that using CI, the confidence ellipsoid induced by $C_{ff}$ contains the one induced by $C({\underline{x}}_{f}-\underline{x})$.

### 5.2. Unbiased Conservative Quantization of Estimates

Applying the Bar-Shalom–Campo formulas (25) and (26) and the covariance intersection Equations (27) and (28) to quantized estimate vectors requires some consideration as naively quantizing the unbiased estimate vectors ${\underline{x}}_{a}$ and ${\underline{x}}_{b}$ does not retain their unbiasedness which in turn leads to biased fused estimates ${\underline{x}}_{f}$. Moreover, quantizing estimate vectors increases their error covariance matrices, which has to be accounted for in order to retain conservativeness of the fusion algorithms. To address these issues we derive a randomized quantizer for unbiased estimate vectors that produces unbiased quantized estimate vectors and provides an upper bound on their error covariance matrices.

We begin by introducing a randomized quantizer $q_{s}:D_{s}\to C_{s}$ with $D_{s}\subset R$ and $C_{s}\subset R$ satisfying $\min(D_{s})$, $\max\left(D_{s}\right)\in C_{s}$, that has the desired unbiasedness property. The quantizer was proposed for estimating quantization in a different but equivalent form in [15] (see also [35,43]) and is defined by

(29) $$\begin{array}{cc} q_{s}\left(\hat{x}\right) & =\mathrm{rd}(\hat{x}+n)\;, \end{array}$$

where $\hat{x}\in R$ is an estimate of a random variable x∈R, rd(·) rounds to the nearest codeword in $C_{s}$, and n∈R is independently uniformly distributed in the closed interval $\left[-\delta_{s}/2,\delta_{s}/2\right]$. The quantizer therefore consists of rounding combined with additive dither [44]. The codebook is given by $C_{s}=\left\{x_{max}-k\delta_{s}\;|\;0\leq k<2^{b}\right\}$, where $x_{max}$ is the maximum codeword, $\delta_{s}=x_{max}/2^{b-1}$ is the increment between adjacent codewords, and *b* is the number of bits required to represent a codeword. The assumption $\min\left(D_{s}\right),\max\left(D_{s}\right)\in C_{s}$ guarantees a quantization error bounded by $\delta_{s}$. It is shown in [15] that $q_{s}$ satisfies

(30) $$\begin{array}{cccc} E(q_{s}\left(\hat{x}\right)) & =E(\hat{x})\;, & C(q_{s}\left(\hat{x}\right)) & \leq C\left(\hat{x}\right)+\delta_{s}^{2}\;, \end{array}$$

that is, it does not add bias and provides an upper bound on the quantization result’s variance. Undesirably, the upper bound is on the variance $C(q_{s}\left(\hat{x}\right))$, not the error variance $C(q_{s}\left(\hat{x}\right)-x)$ and the two quantities coincide only when x is deterministic.

We propose a randomized quantizer $q_{m}:D_{m}\to C_{m}$ for coefficient-wise bounded estimate vectors $\hat{\underline{x}}\in D_{m}\subset R^{n}$ of some random vector $\underline{x}\in R^{n}$ that is an coefficient-wise version of the one given by (29) [34]. Its domain and codebook are the Cartesian products $D_{m}=D_{s}^{n}$ and $C_{m}=C_{s}^{n}$. The quantization process can thus be described by

(31) $$\begin{array}{cc} q_{m}\left(\hat{\underline{x}}\right) & =\mathrm{rd}(\hat{\underline{x}}+\underline{n}) \end{array}$$

where rd(·) now denotes coefficient-wise rounding and $\underline{n}\in R^{n}$ has independent coefficients uniformly distributed in the closed interval $\left[-\delta_{s}/2,\delta_{s}/2\right]$. As an immediate consequence of (30) applied coefficient-wise to $q_{m}\left(\hat{\underline{x}}\right)$, we have the following corollary.

**Corollary** **3.** *Let $q_{m}:D_{m}\to C_{m}$ be as in (31) and $\hat{\underline{x}}\in D_{m}$, then $E\left(q_{m}\left(\hat{\underline{x}}\right)\right)=E\left(\hat{\underline{x}}\right)$ holds.*

Due to rounding and dither, the quantized estimate’s error $q_{m}\left(\hat{\underline{x}}\right)-\underline{x}$ contains additional noise compared to the original estimate’s error $\hat{\underline{x}}-\underline{x}$. Consequently, the known error covariance matrix $C(\hat{\underline{x}}-\underline{x})$ of the original estimate must be adapted to reflect the increased uncertainty. In general, computing the exact covariance matrix of $q_{m}\left(\hat{\underline{x}}\right)-\underline{x}$ is infeasible without knowledge of the distribution (if the distribution of $\hat{\underline{x}}$ was known, the approach in [45] could be used to approximate $C(q_{m}\left(\hat{\underline{x}}\right)-\underline{x})$ arbitrarily well) of $\hat{\underline{x}}$. Therefore, a conservative upper bound for $C(q_{m}\left(\hat{\underline{x}}\right)-\underline{x})$ in a similar vein as (30), is determined.

**Theorem** **8.** *Let $q_{m}:D_{m}\to C_{m}$ be as in (31) and let $\hat{\underline{x}}\in D_{m}$ be an estimate of a random vector $\underline{x}\in R^{n}$, then $C\left(q_{m}\left(\hat{\underline{x}}\right)-\underline{x}\right)⪯C\left(\hat{\underline{x}}-\underline{x}\right)+\delta_{s}^{2}I$ holds.*

**Proof.**  The estimation error covariance matrix after quantization can be expanded into

$$\begin{array}{cc} C(q_{m}\left(\hat{\underline{x}}\right)-\underline{x}) & =C\left(\hat{\underline{x}}-\underline{x}\right)+C\left(q_{m}\left(\hat{\underline{x}}\right)-\hat{\underline{x}}\right)+C\left(\hat{\underline{x}}-\underline{x},q_{m}\left(\hat{\underline{x}}\right)-\hat{\underline{x}}\right)+C\left(q_{m}\left(\hat{\underline{x}}\right)-\hat{\underline{x}},\hat{\underline{x}}-\underline{x}\right) \end{array}$$

by adding and subtracting $\hat{\underline{x}}$. The cross-terms can be shown to be zero by using the definition of $q_{m}$, unbiasedness, and the tower rule to obtain

(32) $$\begin{array}{cc} C(\hat{\underline{x}}-\underline{x},q_{m}\left(\hat{\underline{x}}\right)-\hat{\underline{x}}) & =E\left(E\left(\left(\hat{\underline{x}}-\underline{x}\right){\left(\mathrm{rd}\left(\hat{\underline{x}}+\underline{n}\right)-\hat{\underline{x}}\right)}^{⊤}|\hat{\underline{x}}\right)\right)\;. \end{array}$$

Due to $q_{m}$ being unbiased (also conditionally), $E\left(\mathrm{rd}\left(\hat{\underline{x}}+\underline{n}\right)|\hat{\underline{x}}\right)=\hat{\underline{x}}$ holds. Since $\underline{x}$ and $\underline{n}$ are independent, we have $E\left(\underline{x}\mathrm{rd}{\left(\hat{\underline{x}}+\underline{n}\right)}^{⊤}|\hat{\underline{x}}\right)=E\left(\underline{x}|\hat{\underline{x}}\right)E\left(\mathrm{rd}{\left(\hat{\underline{x}}+\underline{n}\right)}^{⊤}|\hat{\underline{x}}\right)$. Applying these equations to the inner expectation of (32) shows that

$$\begin{array}{cc} C(\hat{\underline{x}}-\underline{x},q_{m}\left(\hat{\underline{x}}\right)-\hat{\underline{x}}) & =E\left(\hat{\underline{x}}{\hat{\underline{x}}}^{⊤}-\hat{\underline{x}}{\hat{\underline{x}}}^{⊤}-E\left(\underline{x}|\hat{\underline{x}}\right){\hat{\underline{x}}}^{⊤}+E\left(\underline{x}|\hat{\underline{x}}\right){\hat{\underline{x}}}^{⊤}\right)=\underline{0}\;, \end{array}$$

as claimed. As $C(\hat{\underline{x}}-\underline{x})$ is known, it only remains to bound $C(q_{m}\left(\hat{\underline{x}}\right)-\hat{\underline{x}})$. Since $q_{m}$ is (conditionally) unbiased and $\underline{n}$ has independent coefficients,

$$\begin{array}{cc} E\left(\left(q_{m}{\left(\hat{\underline{x}}\right)}^{i}-{\hat{\underline{x}}}^{i}\right)\left(q_{m}{\left(\hat{\underline{x}}\right)}^{j}-{\hat{\underline{x}}}^{j}\right)|\hat{\underline{x}}\right) & =E\left(q_{m}{\left(\hat{\underline{x}}\right)}^{i}-{\hat{\underline{x}}}^{i}|\hat{\underline{x}}\right)E\left(q_{m}{\left(\hat{\underline{x}}\right)}^{j}-{\hat{\underline{x}}}^{j}|\hat{\underline{x}}\right)=0 \end{array}$$

follows for i≠j, which by the tower rule and unbiasedness results in

$$\begin{array}{c} C\left(q_{m}{\left(\hat{\underline{x}}\right)}^{i}-{\hat{\underline{x}}}^{i},q_{m}{\left(\hat{\underline{x}}\right)}^{j}-{\hat{\underline{x}}}^{j}\right)=E\left(\left(q_{m}{\left(\hat{\underline{x}}\right)}^{i}-{\hat{\underline{x}}}^{i}\right)\left(q_{m}{\left(\hat{\underline{x}}\right)}^{j}-{\hat{\underline{x}}}^{j}\right)\right)=0 \end{array}$$

for i≠j. Furthermore, it holds that $|q_{m}{\left(\hat{\underline{x}}\right)}^{i}-{\hat{\underline{x}}}^{i}|\leq \delta_{s}$ and thus $C\left(q_{m}{\left(\hat{\underline{x}}\right)}^{i}-{\hat{\underline{x}}}^{i}\right)\leq \delta_{s}^{2}$ for i=1,…,n. Combined, this means that $C\left(q_{m}\left(\hat{\underline{x}}\right)-\hat{\underline{x}}\right)⪯\delta_{s}^{2}I$. The claimed upper bound then follows immediately. □

The upper bound given above is fast to compute and does not require any knowledge of the distribution of either $\underline{x}$ or $\hat{\underline{x}}$, at the cost of being overly conservative, particularly if a component of $\hat{\underline{x}}$ is concentrated between two codewords or for large $\delta_{s}$.

### 5.3. Quantized Optimal Fusion and Covariance Intersection

We are now in the position to formulate a process that allows to apply the Bar-Shalom–Campo formulas or covariance intersection to quantized estimates and covariance matrices while retaining unbiasedness and conservativeness of said fusion methods. The proposed approach is as follows:

1. Quantize the estimates ${\underline{x}}_{a}$ and ${\underline{x}}_{b}$ so that the quantization results remain unbiased. Account for the potential increase in uncertainty due to the quantization process. Both goals are achieved by employing the unbiased, conservative estimate quantizer introduced in the previous subsection.
2. Quantize the error covariance matrices of the quantized estimates conservatively. This is done using either the quantizer from Section 4.1 or the one from Section 4.2.
3. Apply the Bar-Shalom–Campo formulas or covariance intersection to the quantized estimates and quantized error covariance matrices. Since the quantized estimates are unbiased and the quantized error covariance matrices are conservative the fusion result will also be unbiased and conservative.

In the following, the above process using CI in conjunction with either the diagonal dominance (DD)-based quantizer from Section 4.1 or the modified Cholesky (MC) decomposition-based quantizer from Section 4.2 will be referred to as DD-CI and MC-CI, respectively. The methods obtained by replacing CI with the optimal (OPT) fusion formulas in the DD-CI and MC-CI methods will be referred to as DD-OPT and MC-OPT.

## 6. Results and Discussion

The total quantization errors of the proposed covariance matrix quantizers are evaluated using randomly selected covariance matrices. In addition, the performance of DD-CI/OPT and MC-CI/OPT relative to CI/optimal fusion is evaluated by applying the methods to randomly generated data. Finally, the DD-CI and MC-CI approaches are evaluated in a decentralized 2D target tracking scenario.

### 6.1. Evaluation of the Covariance Quantizers

**Figure 3:**
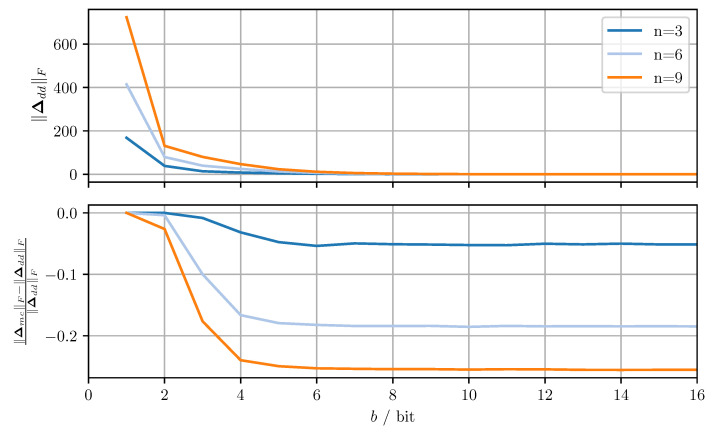
Average Frobenius norm of the quantization error matrix $\Delta_{dd}$ of the diagonal dominance-based quantization approach (**top**), and the relative improvement achieved by the modified Cholesky-based quantization approach (**bottom**) for varying dimensions *n* and bits per codeword *b*.

The two proposed covariance matrix quantizers are applied to independent random covariance matrices. The random matrices are generated as $X=LL^{⊤}$ where $L\in R^{n\times n}$ has zero-mean, normally distributed elements with variance one. The Frobenius norms of the resulting quantization error matrices are averaged over all samples. Figure 3 shows the averaged Frobenius norm of the diagonal dominance based quantizer and the relative improvement in average norm achieved by the modified Cholesky decomposition based quantizer for varying numbers of bits per codeword *b* and matrix dimensions *n*. For the codebooks, $x_{max}=50.0$ was used and 10,000 samples were included in each average.

The quantization error increases monotonically as *b* decreases with larger *n* leading to stronger deterioration of performance. The dependency on *b* is a result of the quantization resolution decreasing exponentially for decreasing *b*. The dependency on *n* is due to the fact that for increasing *n* the quantization error per element remains roughly the same, whereas the number of matrix elements increases quadratically. Therefore the off-diagonal quantization error increases for increasing *n*. The shift applied to the diagonal elements to ensure conservativeness must then grow larger with increasing *n*, thereby increasing the diagonal quantization error. The relative improvement in average norm can be seen to be approximately constant over *b*, the notable exception being b≤2 where there is little to no improvement. This behavior can be understood by considering the limiting case of b=1. In said case, each element of the quantized matrix is either zero or the maximum codeword of the respective codebook. Since the off-diagonal elements are identical in both approaches and the diagonal elements are always rounded up, the quantized matrix and thus the quantization error are identical in both approaches.

### 6.2. Evaluation of Quantized Optimal Fusion and Quantized Covariance Intersection

The test data for this evaluation are generated by first drawing a zero mean Gaussian random vector $\underline{x}\in R^{n}$ with covariance matrix $C(\underline{x})=I$. This vector represents the ground truth. Then, a random vector $\underline{z}={\left[{\underline{z}}_{a}^{⊤},{\underline{z}}_{b}^{⊤}\right]}^{⊤}\in R^{2n}$ is drawn from a zero mean Gaussian distribution with covariance matrix $LL^{⊤}$, where $L\in R^{2n\times 2n}$ has zero mean Gaussian elements with variance one. Finally, two correlated estimates ${\underline{x}}_{a}=\underline{x}+{\underline{z}}_{a}$ and ${\underline{x}}_{b}=\underline{x}+{\underline{z}}_{b}$ of $\underline{x}$ are computed. Optimal fusion, covariance intersection and their quantized versions are applied to the estimates ${\underline{x}}_{a}$ and ${\underline{x}}_{b}$ using their known conditional (cross-)covariance matrices

$$\begin{array}{cc} C({\underline{x}}_{a}|L) & =C\left(\underline{x}\right)+C\left({\underline{z}}_{a}|L\right)\\ C({\underline{x}}_{b}|L) & =C\left(\underline{x}\right)+C\left({\underline{z}}_{b}|L\right)\\ C({\underline{x}}_{a},{\underline{x}}_{b}|L) & =C\left(\underline{x}\right)+C\left({\underline{z}}_{a},{\underline{z}}_{b}|L\right). \end{array}$$

The mean squared errors ${\mathrm{MSE}}_{f}=E(∥{\underline{x}}_{f}-\underline{x}{∥}^{2})$, where ${\underline{x}}_{f}$ is the fused estimate with *f* indicating the fusion approach, are computed by repeatedly generating test data, applying the fusion approaches, and averaging the squared Euclidean norm of the resulting estimate errors. The mean traces ${\mathrm{MTR}}_{f}=E\left(\mathrm{tr}\left(C_{ff}\right)\right)$ of the error covariance estimates $C_{ff}$, where *f* indicates the fusion approach, are also computed using averaging. If any quantization operation fails for any of the test data, the computed MSE and averaged trace are discarded.

**Figure 4:**
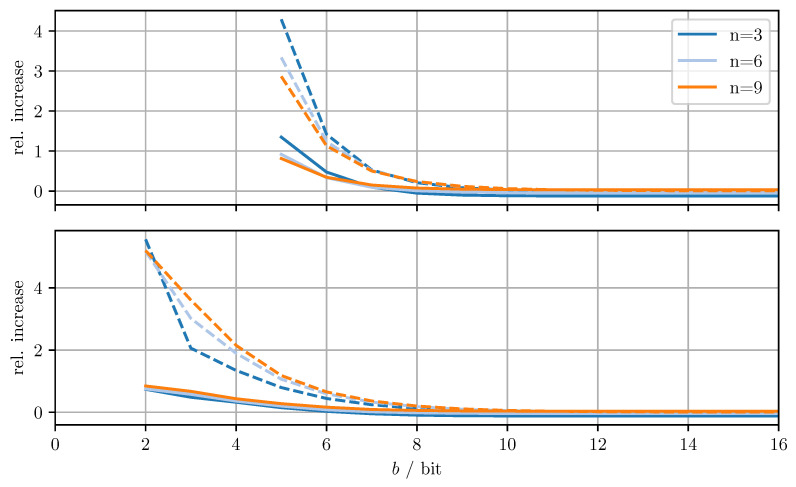
Relative increase of actual MSE (solid)/averaged trace (dashed) of DD-OPT with respect to OPT for varying dimensions *n* and bits per codeword *b*. Top row with quantized estimate vector and quantized error covariance, bottom row only with quantized error covariance.

**Figure 5:**
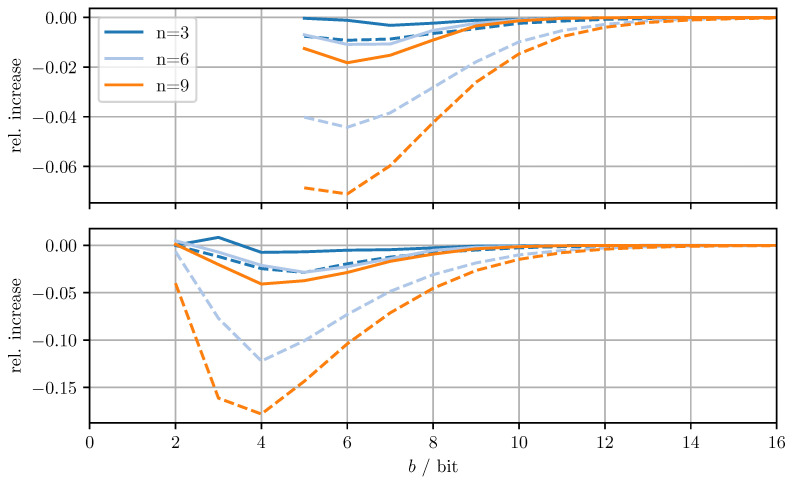
Relative increase of actual MSE (solid)/averaged trace (dashed) of MC-OPT with respect to DD-OPT for varying dimensions *n* and bits per codeword *b*. Top row with quantized estimate vector and quantized error covariance, bottom row only with quantized error covariance.

**Figure 6:**
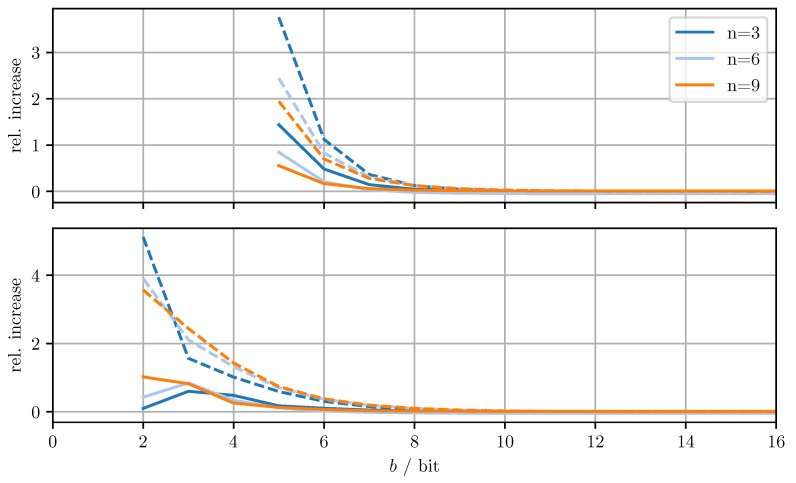
Relative increase of actual MSE (solid)/averaged trace (dashed) of DD-CI with respect to CI for varying dimensions *n* and bits per codeword *b*. Top row with quantized estimate vector and quantized error covariance, bottom row only with quantized error covariance.

**Figure 7:**
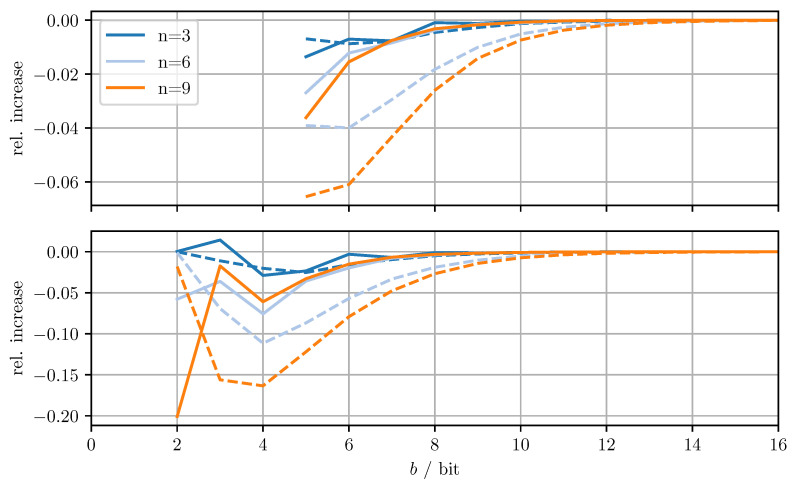
Relative increase of actual MSE (solid)/averaged trace (dashed) of MC-CI with respect to DD-CI for varying dimensions *n* and bits per codeword *b*. Top row with quantized estimate vector and quantized error covariance, bottom row only with quantized error covariance.

Figure 4 shows the relative increase $\left({\mathrm{MSE}}_{DD-OPT}-{\mathrm{MSE}}_{OPT}\right)/{\mathrm{MSE}}_{OPT}$ of the MSE and the relative increase $\left({\mathrm{MTR}}_{DD-OPT}-{\mathrm{MTR}}_{OPT}\right)/{\mathrm{MTR}}_{OPT}$ of the averaged trace when using DD-OPT instead of OPT. Figure 5 shows the relative increase $\left({\mathrm{MSE}}_{MC-OPT}-{\mathrm{MSE}}_{DD-OPT}\right)/{\mathrm{MSE}}_{DD-OPT}$ of the MSE and the relative increase of the averaged trace $\left({\mathrm{MTR}}_{MC-OPT}-{\mathrm{MTR}}_{DD-OPT}\right)/{\mathrm{MTR}}_{DD-OPT}$, when using MC-OPT instead of DD-OPT. Figure 6 and Figure 7 show the same quantities but compare DD-CI to CI and MC-CI to DD-CI, respectively. In all figures, varying dimensions *n* and numbers of bits per codeword *b* are considered both with and without quantizing estimate vectors. The results are obtained by averaging over 1000 independent trials and using $x_{max}=65.0$ for the codebooks $C_{m}$, $C_{d}$, and $C_{o}$.

From Figure 4, it can be seen that for DD-OPT the increase of the estimate MSE and of the averaged trace of the error covariance estimate is moderate except for small *b*. Moreover, the increase in the averaged trace is larger than the increase in MSE for all *n* and *b*. This is to be expected, since the quantization process retains conservativeness. The dimensionality of the test data has varying influence on the performance, depending on *b* and on whether quantized estimates are being used. Using quantized estimate vectors is seen to adversely affect performance. In fact, when quantizing the estimate vectors the error covariance quantization fails below five bits per codeword, due to the excessively inflated error covariance estimate produced by the estimate quantization. Figure 5 shows that the MC-OPT approach performs better than the DD-OPT approach in terms of the average trace of the error covariance estimate and in most cases also in terms of actual MSE. Larger *n* leads to larger improvements. The improved performance in terms of average trace is guaranteed by the theoretical results from Section 4.2. Note that there is no guarantee that the actual MSE reduces if the error covariance matrices are quantized more accurately. It can also be seen that there is an optimal number of bits per codeword and that for small *b* there is little to no improvement. The latter result is due to the phenomenon discussed in Section 6.1. The results for CI, DD-CI, and MC-CI displayed in Figure 6 and Figure 7 exhibit the same general behavior as the results for OPT, DD-OPT, and MC-OPT discussed above, also in terms of theoretical guarantees. One notable difference is the relative improvement of actual MSE. In contrast to optimal fusion, the improvement does not seem to diminish for large *n* and small *b*.

### 6.3. Evaluation of Quantized Covariance Intersection in 2D Tracking Scenario

This evaluation scenario considers two sensor nodes that cooperatively track an object. The object is characterized by a discrete-time (nearly) constant acceleration model

$$\begin{array}{ccc} {\underline{x}}_{k+1}=\left[\begin{array}{cc} A & 0\\ 0 & A \end{array}\right]\;{\underline{x}}_{k}+{\underline{w}}_{k}\;, & \; & {\underline{w}}_{k}∼N\left(\underline{0},\left[\begin{array}{cc} Q & 0\\ 0 & Q \end{array}\right]\right)\;, \end{array}$$

affected by the zero-mean white Gaussian noise term ${\underline{w}}_{k}\in R^{6}$. The six-dimensional state ${\underline{x}}_{k}\in R^{6}$ consists of position, velocity, and acceleration in both the $x^{1}$- and $x^{2}$-direction. The corresponding matrices of the process model are given by

$$\begin{array}{ccc} A=\left[\begin{array}{ccc} 1 & \tau & \frac{1}{2}\tau^{2}\\ 0 & 1 & \tau \\ 0 & 0 & 1 \end{array}\right]\;, & \; & Q=0.5\left[\begin{array}{ccc} \frac{1}{20}\tau^{5} & \frac{1}{8}\tau^{4} & \frac{1}{6}\tau^{3}\\ \frac{1}{8}\tau^{4} & \frac{1}{3}\tau^{3} & \frac{1}{2}\tau^{2}\\ \frac{1}{6}\tau^{3} & \frac{1}{2}\tau^{2} & \tau \end{array}\right]\;, \end{array}$$

where τ>0 is the time step [46]. For the Monte Carlo simulation with 1000 runs, the initial states ${\underline{x}}_{0}$ are drawn from

$$\begin{array}{c} {\underline{x}}_{0}∼N\left(\left[\begin{array}{c} 0\\ 0\\ 0.2\\ 0\\ 0\\ 0.3 \end{array}\right],\left[\begin{array}{cccccc} 0.5 & 0 & 0 & 0 & 0 & 0\\ 0 & 0.1 & 0 & 0 & 0 & 0\\ 0 & 0 & 0.05 & 0 & 0 & 0\\ 0 & 0 & 0 & 0.5 & 0 & 0\\ 0 & 0 & 0 & 0 & 0.1 & 0\\ 0 & 0 & 0 & 0 & 0 & 0.05 \end{array}\right]\right)\;. \end{array}$$

Two sensor nodes *a* and *b* are simulated that observe projections of position and velocity according to

$$\begin{array}{c} {\underline{z}}_{k}^{a,b}=\left[\begin{array}{cccccc} \cos(\theta^{a,b}) & \sin(\theta^{a,b}) & 0 & 0 & 0 & 0\\ 0 & 0 & \cos(\theta^{a,b}) & \sin(\theta^{a,b}) & 0 & 0 \end{array}\right]\;{\underline{x}}_{k}+{\underline{v}}_{k}^{a,b} \end{array}$$

with $\theta^{a}=\frac{\pi }{4}$, $\theta^{b}=-\frac{\pi }{8}$. The zero-mean white Gaussian measurement noise terms ${\underline{v}}_{k}^{a}\;,{\underline{v}}_{k}^{b}\in R^{2}$ have the covariance matrix

$$\begin{array}{cc} R^{a}=0.5\left[\begin{array}{cc} 1 & 0\\ 0 & 0.1 \end{array}\right]\;, & \;R^{b}=0.8\left[\begin{array}{cc} 1 & 0\\ 0 & 0.5 \end{array}\right]\;, \end{array}$$

respectively. Each sensor node uses a Kalman filter to compute estimates for 50 time steps. Sensor node *a* transmits its state and error covariance estimate to sensor node *b* at every 5th time step. Prior to transmission, it quantizes the estimates with the proposed method and codebook parameter $x_{max}=30.0$. Node *b* fuses its own estimate with the received one by employing CI. Every 11th time step, sensor node *b* quantizes and transmits its state and error covariance estimate to node *a*, which again fuses it with its own estimate using CI. The receiving node in both cases reinitializes its own estimate with the fusion result.

**Figure 8:**
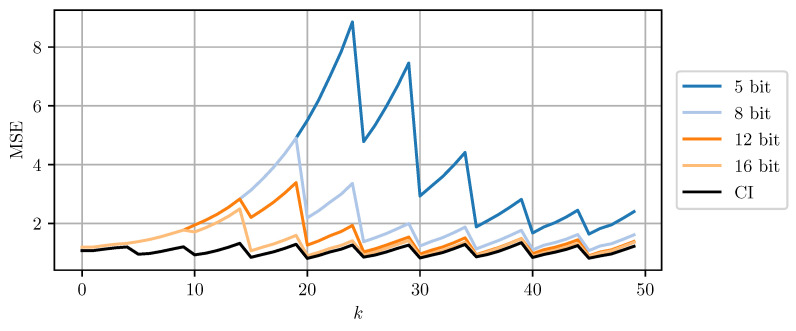
The estimate MSE of sensor node b plotted over time step *k* for varying bits per codeword using DD-CI. ‘CI’ indicates the use of a 64 bit floating point representation for each scalar.

**Figure 9:**
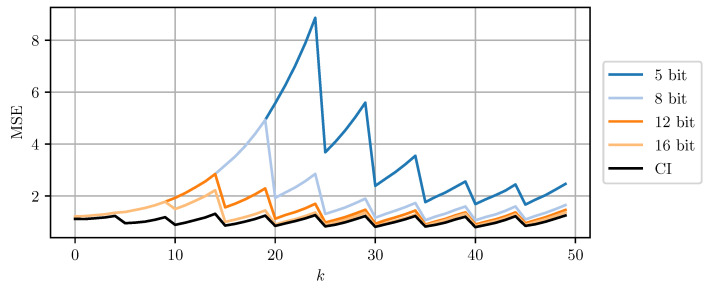
The estimate MSE of sensor node b plotted over time step *k* for varying bits per codeword using MC-CI. ‘CI’ indicates the use of a 64 bit floating point representation for each scalar.

Figure 8 and Figure 9 compare DD-CI and MC-CI using different numbers of bits per codeword, against CI using 64-bit floating point numbers. The results for higher numbers of bits per codeword are close to the estimates obtained through CI with 64-bit floats. However, even a 5-bit quantization still yields reasonable results. Quantization using less than 5 bits per codeword leads to too conservative bounds on the error covariance matrices that cannot be encoded using the given codebook. The estimate MSE exhibits an initial transient peak. It is here, that the improved performance of MC-CI over DD-CI can be observed most clearly.

## 7. Conclusions

Available bandwidth and energy budget can be limiting factors for the data transmission capabilities of interconnected sensor systems. Algorithms for decentralized information fusion in networks require the exchange of estimates and, in some cases, covariance matrices. If covariance matrices need to be transmitted, they dominate the amount of transmitted data. In this paper, we have proposed two methods for the conservative quantization of covariance matrices, a method for the unbiased conservative quantization of estimates, and have applied them to optimal fusion and covariance intersection. The presented quantization approaches retain unbiasedness and conservativeness of the considered fusion methods while reducing the amount of data that must be transmitted. We have empirically demonstrated the effectiveness of the proposed covariance quantization methods, individually and in conjunction with fusion methods. Further improvements in performance could be achieved by using varying, possibly data-dependent quantization resolutions for subsets of the coefficients of the considered covariance matrices. Moreover, the proposed quantization schemes can also be applied to other sensor fusion algorithms like inverse covariance intersection. For future work, theoretical results concerning the convergence behavior of state and covariance estimates when using quantized data in a decentralized setting are of interest. Conservative vector quantization for covariance matrices is also worth consideration.

## Data Availability

Not applicable.

## Supplementary Materials

The following are available online at https://www.mdpi.com/article/10.3390/s21093059/s1, Python implementations of the quantization approaches.
